# Testicular arteries anatomy applied to fowler-sthephens surgery in high undescended testis – a narrative review

**DOI:** 10.1590/S1677-5538.IBJU.2021.99.11

**Published:** 2021-05-10

**Authors:** Tatiana C. Benzi, Natasha T. Logsdon, Francisco J. B. Sampaio, Luciano Alves Favorito

**Affiliations:** 1 Universidade do Estado do Rio de Janeiro Unidade de Pesquisa Urogenital Rio de Janeiro RJ Brasil Unidade de Pesquisa Urogenital - Universidade do Estado do Rio de Janeiro - Uerj, Rio de Janeiro, RJ, Brasil

**Keywords:** Cryptorchidism, Anatomy, Testis

## Abstract

**Objectives:**

In this review we will describe the testicular vessels anatomy and the implications of these vessels in surgical treatment of high undescended testis.

**Material and Methods:**

We performed a narrative review of the literature about the role of the testicular arteries anatomy in the treatment of high undescended testis. We also studied two human testes to illustrate the testicular vascularization.

**Results:**

Each testis is irrigated by three arteries: testicular artery (internal spermatic artery), a branch of the right aorta; deferential artery (vasal artery), a branch of the inferior vesicle artery that originates from the anterior trunk of internal iliac artery and cremasteric artery (external spermatic artery), a branch of the inferior epigastric artery. There are important communications among the three arteries with visible anastomotic channels between the testicular and deferential arteries.

**Conclusions:**

Laparoscopic transection of the testicular vessels by dividing the spermatic vessels (Fowler-Stephens surgery) is safe in patients with high abdominal testis due to the great collateral vascular supply between testicular, vasal and cremasteric arteries; also, two-stage Fowler-Stephens orchiopexy appears to carry a higher rate of success than the single stage approach.

## INTRODUCTION

Cryptorchidism is the consequence of lack or insufficiency of the process of testicular descent taking place during fetal life (1). Undescended testis is one of the most common genital diseases identified at birth and the most common surgical problem in pediatric urology. The main justification for treatment is to reduce the increased risks of infertility, testicular malignancy, and/or torsion/trauma as well as inguinal hernia associated with the undescended testis (2).

Studies show that, in Europe, the percentage of individuals with cryptorchidism ranges from 2% to 8%, 6% in the United Kingdom (3). According to some papers, 2% to 8% of full-term newborns have one or both testes not descended at birth (4, 5). Goel (6) reports a percentage between 1% and 4% in full-term newborns and 30% in preterm infants.

Approximately 20% of the cryptorchid testes are non-palpable (7, 8). A subset of these patients may represent a surgical challenge once identifying the correct location of the undescended testis is important to select the proper surgical treatment. The non-palpable testis may be intra-abdominal and thus associated with either an open or closed internal inguinal ring. Alternatively, some testes may migrate back and forth across the internal inguinal ring thus being palpable on first exam but not on subsequent exam and are referred as peeping testes. The testis may also be completely absent or atrophic (9).

In this review we will describe the testicular vessels anatomy and the implications of these vessels in surgical treatment of high undescended testis.

## MATERIAL AND METHODS

In this study we carried out a review about the role of the testicular arteries anatomy in the treatment of high undescended testis. We analyzed papers published in the past 60 years on Pubmed, Embase and Scielo databases searching by key the following expressions: “Undescended testis”, “Cryptorchidism”, “Fowler-Stephens Surgery”, “High undescended testis”, “Testicular artery”, “Cremasteric artery” and “vasal artery”. In this review we found several papers, but we included only papers published in English and excluded case reports, editorials, and opinions of specialists.

We also studied two human fetuses (one with 20 weeks post conception and the second with 32 weeks post conception) to illustrate the testicular vascularization. The fetuses were well preserved and have been demised due to spontaneous or induced abortion. The gestational age of the fetuses was determined in weeks post conception (WPC) according to the foot-length criterion, which is currently considered the most acceptable parameter to estimate the fetal gestational age (10–12).

**Figure 1:**
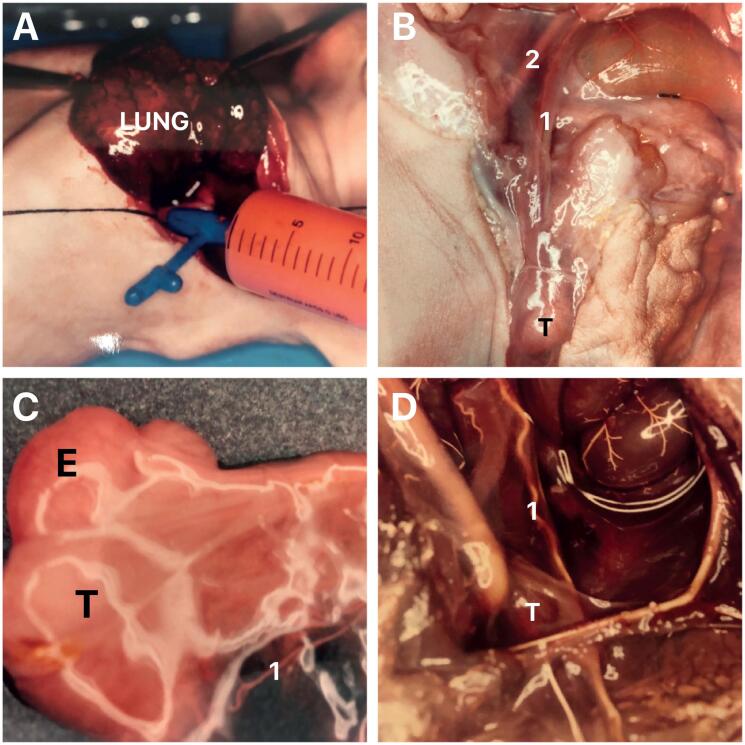
The figure shows the two human fetuses studied with the microvascular silicone rubber resin technique. A) The figure shows a fetuses with 32 weeks post-conception injected through the thoracic aorta with a microvascular silicone rubber red resin to fill in the arterial tree; B) After the injection the fetuses were dissected under low magnification using a surgical microscope and in this figure we can observe the right testis (T) in scrotal position and the testicular artery (1) and the external iliac artery (2); C) In the same fetuses the testis (T) was resected and we can observe the vassal artery (1), E- Epididymis and D) In this figure we can observe a fetus of the 2nd gestational trimester with the testis (T) in abdominal position, near the internal ring injected with the with rubber silicone resin, the testicular artery (1) is easily identified.

After measuring the fetuses, microvascular silicone rubber resin (red or white) was injected through the thoracic aorta to fill the arterial tree. The testes were identified and the vessels were dissected under low magnification using a surgical microscope. (Figure-1)

## RESULTS

In 2014, the American Urologic Association (AUA) guidelines for evaluation and treatment of cryptorchidism were published and recommended that: 1) at initial evaluation a complete gestational history of all boys suspected of cryptorchidism must be obtained; 2) the testicles should be palpated at each recommended well-child visit for appropriate quality and position; 3) all infants who are found to have cryptorchidism at birth and who do not have spontaneous descent by age of 6 months (corrected for gestational age) must be referred to a surgeon for appropriate evaluation; 4) all boys with a possible new diagnosis of acquired cryptorchidism after 6 months of corrected gestational age be referred for possible surgical correction; 5) any phenotypic male newborns with bilateral non-palpable testes be referred for evaluation of possible disorder of sexual development (DSD); 6) ultrasound and other imaging studies (which are rarely sensitive diagnostic tools) not be ordered prior to referral to a surgical specialist; 7) severe proximal hypospadias and cryptorchidism alert the provider to assess for DSD; 8) a boy who is found to have bilateral non-palpable testes and who does not have congenital adrenal hyperplasia, Müllerian inhibiting substance, or other additional hormones be tested to evaluate for anorchia; and 9) at least annual physical exams be used to assess for secondary ascent in boys found to have retractile testicles (2).

Current guidelines recommend orchidopexy during the first year of life; however, this seems not to be implemented in practice. Currently, only a small proportion of boys with undescended testicles are operated upon during their first year of life. The level of knowledge in attending physicians remains in need of improvement (13). The recommended age for orchidopexy was reduced to below 1 year based on findings of germ cell loss in the undescended testicle at 1 to 2 years of age and findings that orchidopexy performed at 9 months compared with 3 years had a more significant beneficial effect on the growth of the previously undescended testicles (2).

The purpose of surgical treatment is the placement of the testis in the scrotum without atrophy or recurrence (14). In case the testicles are not palpable, laparoscopic, or open surgical exploration is necessary, allowing to identify the morphology and anatomical locations of the testis, deferens and testicular vessels to select the most appropriate surgical technique for treatment. Laparoscopy has an advantage over open surgery as it better identifies the anatomy, viability, and location of the impalpable testis (15).

### Testicular Arteries

Regardless of the surgical technique used, preservation of an adequate arterial supply for the testis is crucial for successful orchiopexy to ensure normal testicular size and texture. The anatomy of testicular arteries is very important in the success of the surgical procedures.

The vascularization of the XX and XY gonads is a highly dynamic process. The XY gonad recruits and patterns vasculature by a novel remodeling mechanism beginning with the breakdown of an existing mesonephric vessel. The gonads are highly vascularized and they present an important collateral circulation since the beginning of development (16).

**Figure 2:**
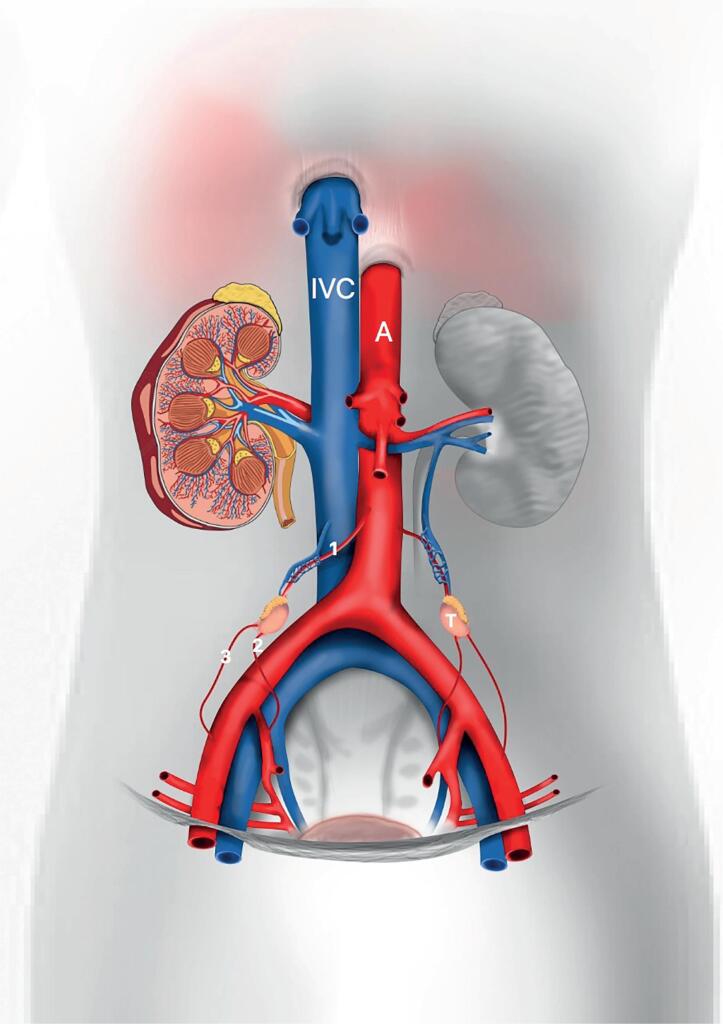
Schematic drawing showing the testicular vascularization during the human fetal period. We can observe the testis situated in abdominal position and the three arteries that irrigated the gonad, 1- testicular artery, 2- vassal artery, 3- cremasteric artery, A- Aorta artery and IVC- Inferior vena cava.

Each testis is irrigated by three arteries: testicular artery (internal spermatic artery), a branch of the right aorta; deferential artery (vasal artery), a branch of the inferior vesicle artery that originates from the anterior trunk of internal iliac and cremasteric arteries (external spermatic artery), a branch of the inferior epigastric artery (Figure-2). These three arteries penetrate the organ in the mediastinal region providing ample communication (17–19).

**Figure 3:**
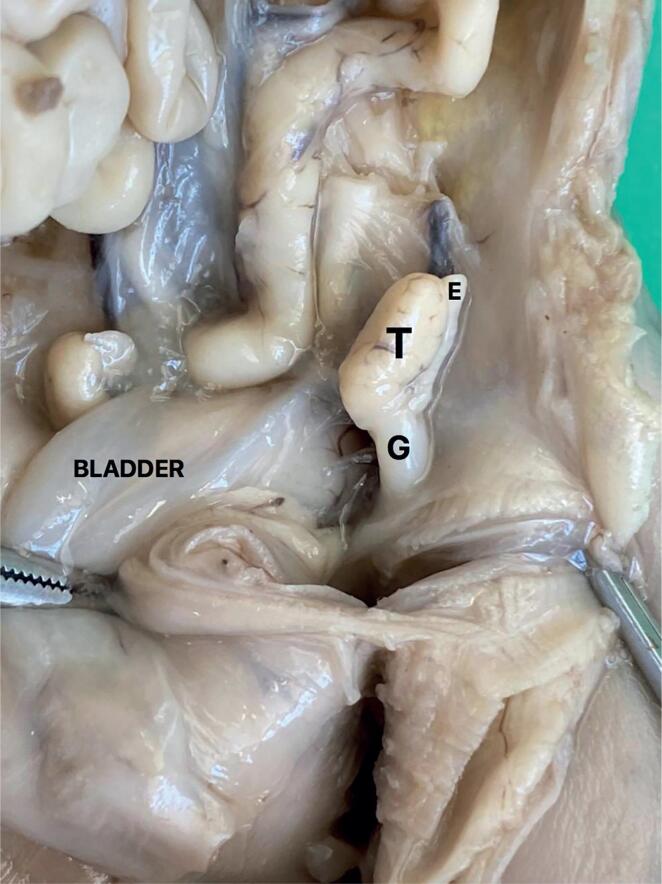
The figure shows a male fetus with 15 weeks post-conception with both testes situated in the abdomen. The abdominal wall was dissected to show the position of the left testis (T) above the internal ring, G- gubernaculum testis and E- Epididymis.

**Figure 4:**
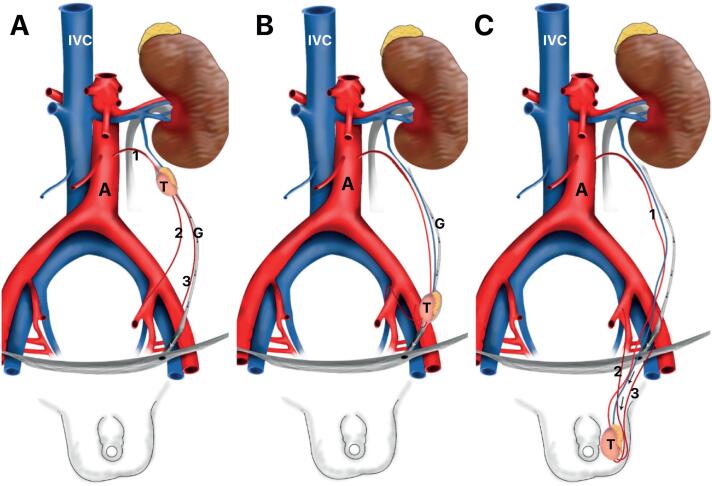
Schematic drawing showing the steps of testicular migration and the testicular arteries. A) Testis (T) in high abdominal positon, we can observe that the testicular artery (1) is short and the vasal artery (2) and cremasteric artery (3) are longer; B) Testis in abdominal position near the internal inguinal ring and C) Testis in scrotal position, G- gubernaculum, A- Aorta Artery and IVC- Inferior Vena Cava.

Modifications in the position and anatomy of the testicular arteries during the process of testicular migration needs to be related. The testicular migration has two phases: the abdominal and the inguinoscrotal stages (20–22). During the abdominal stage testes migrate from the abdomen to the internal inguinal ring. This process begins around the 8th WPC and lasts until the 15th WPC. The second stage (inguinoscrotal stage) is the transition of the testes through the inguinal canal until their definitive arrival in the scrotum (19, 23). Distally, the gubernaculum approaches the inguinal region (Figure-3). During this stage, after the testes crosses the external inguinal ring the gubernaculum migrates across the pubic region to reach the scrotum (24, 25). The passage of the testis through the inguinal canal occurs very quickly, between 21 and 25 WPC, and the testicular migration process completes around the 30th week post conception (26). In Figure 4 we show the chronology of testicular migration and the arterial branches of the testis when the testis is in high abdominal position, near the inguinal ring and in scrotum.

In an interesting anatomical study in cadavers and undescended testis, using the injection-corrosion casting technique, it was demonstrated for all testes studied, including the undescended ones, had testicular, they presented deferential and cremasteric arteries (27). This study shows that exists an important communication among the three arteries with visible anastomotic channels between the testicular and deferential arteries. This important paper suggested that the concept of high ligation of the testicular artery is valid to preserve vascularity to the testis during the vascular transection orchiopexy (27).

**Figure 5:**
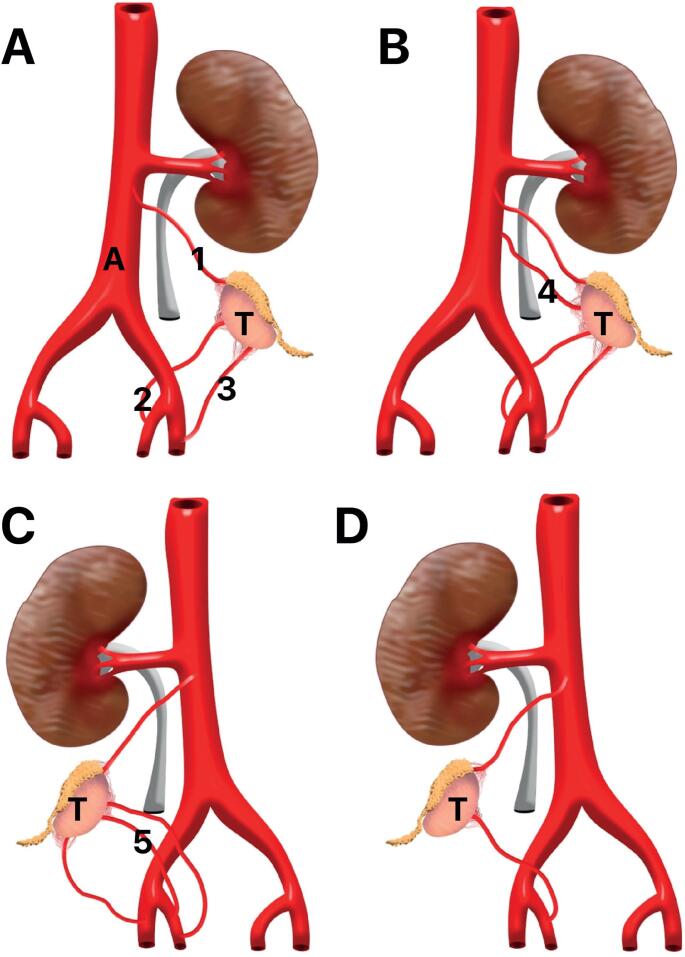
Testicular vascularization variations in fetuses with the testis in abdominal position. A) Schematic drawing showing the most frequent arterial pattern of testicular vascularization - Testis (T) has three arteries: Testicular (1), Deferential (2) and Cremasteric (3); B) Schematic drawing showing testicular vascularization with four arteries, we can observe an anatomical variation - presence of a second testicular artery (4) originated at the aorta artery; C) Schematic drawing showing testicular vascularization with four arteries, we can observe an anatomical variation - presence of a second vasal artery (5) originated at the internal iliaca artery and D) Schematic drawing showing testicular vascularization with two arteries only, the testicular artery and vasal artery, the cremasteric artery is not present, A- Aorta artery, T- Testis.

In an experimental study with 32 human fetuses (64 testis), the authors studied the testicular vascularization in fetuses with the testis in abdominal position (28). The authors demonstrated 23.4% of testes have 2 arteries (1 testicular and 1 deferential), 71.9% have 3 arteries (1 testicular, 1 deferential and 1 cremasteric) and only 4.7% of the testes had 4 arteries (1 testicular, 2 deferentials and 1 cremasteric or 2 testiculars, 1 deferential and 1 cremasteric). This important paper concluded that the fetal testicle is always irrigated by at least 3 arteries (testicular, cremasteric and deferential) in almost 80% of cases and in the other 20% of the cases there were only 2 arteries irrigating the abdominal testis, an important information to the surgery of high undescended testis. Figure-5 shows the anatomical variations showed in this important paper.

Knowledge about the anatomy of the veins that drain the testes is clinically important. The testes are drained by the pampiniform venous plexus, which in the region of the deep inguinal ring originates the testicular veins. The left testicular vein opens into the left renal vein and the right testicular vein opens directly into the inferior cava vein (29).

### Fowler-Stephens Surgery

Today, the laparoscopic approach to the inguinal and intra-abdominal non-palpable testicles is the first choice of most surgeons (30), it allows an accurate identification of testicular location, spermatic funiculus characteristics and epididymis. It is one of the safest methods for the confirmation of unilateral cryptorchidism, depending directly on the experience of the surgeon and the availability of specialized material for its realization in young children (31).

Orchidodopexy in patients with intra-abdominal testicles is applied using one out of three surgical techniques: conventional orchiopexy, laparoscopic orchiopexy, Fowler-Stephens orchiopexy at one stage, and Fowler-Stephens orchiopexy in two stages. In the three types of techniques, the surgery consists of locating the testis, dissectising it near the spermatic cord, obtaining free tension, and then repositioning it near the scrotum. When performed open, a medial inguinal incision is made from the anterosuperior iliac spine to the external oblique fascia allowing exploration of the peritoneal cavity. The advantages of performing these techniques by laparoscopy include improved visualization, extensive vascular dissection capacity at vessel origin, lower morbidity, and ability to create a medial inner ring for the lower epigastric vessels, also performing a direct course to the scrotum (32).

**Figure 6:**
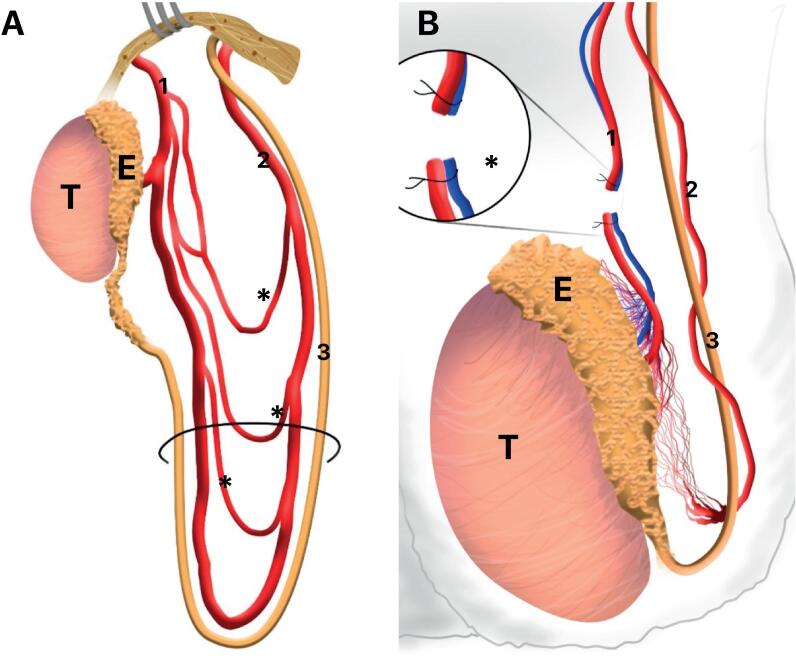
Testicular arteries pattern showed in the original Fowler-Stephens paper (x). A) Schematic drawing showing the vascular supply of the testis, we can observe an important collateral circulation (*) through the Testicular artery (1) and deferential artery (2), 3- Vas deferens; B) Schematic drawing showing the place of ligature of testicular vessels (*) in Fowler-Stephens surgery, T- testis, E- Epididymis.

Fowler and Stephens in 1959 show the vascular supply of the testes applied in the surgical treatment of high undescended testis and showed an important collateral circulation through the deferential artery and the cremasteric artery (33). In this important paper the authors showed some points where the arteries have communications (Figure-6).

Fowler-Stephens orchiopexy, both in one and two stages, is adopted when the testis is located high and the testicular vessels are too short to be fixed in the scrotum. The vessels are ligated and the blood supply to the testis preserved via collateral circulation and proceeding with the repositioning and fixation of the testis (34). In the one-time Fowler-Stephens technique, the ligature and fixation of the testis are performed in a single surgical time, while in the two-stage, the first phase of the surgical treatment is performed to ligate the testicular vessels and in a second surgery, three to six months after the first surgical time, the testis is repositioned and fixed to the scrotal pouch (35, 36). Whenever possible, primary orchiopexy is adopted, which has the highest efficiency rate. Comparing the two Fowler-Stephens techniques, the two-step technique has higher success rates (36, 37).

According to Igarashi (38), in a study with seventy-two impalpable testes diagnosed in 68 patients, it is recommended starting inguinal exploration for impalpable testis, considering the relatively low incidence of high abdominal testis. When an extra-abdominal testis is not detected, transinguinal laparoscopic scanning should be indicated.

Geuvbashian et al. (39) reported in their study a high success rate after surgical management of non-palpable testicles regardless of technique. The rate of testicular atrophy is similar in both artery preservation and SF for non-palpable testis. There is no significant difference between 1- and 2-stage FSO. In 2019, Abouheba et al. (40), evaluated the short-term clinical outcome of the new Shehata laparoscopic tensile stretching technique for abdominal testis. They concluded that this new Shehata technique is safe, easy and convenient. They observed that neither the internal hernia complicated the traction period, nor testicular atrophy (due to undue tension) complicated traction or follow-up periods. Thus, they reported that it is a good alternative to staged Fowler-Stephens orchiopexy, which entails a risky division of testicular vessels.

In an interesting study with patients undergoing a second-stage FS procedure the authors photographed prior to pexy of the testis in the Dartos pouch. The photographs were evaluated for the extent of vascular collateralization between gubernacular, deferential and the ligated spermatic artery, and concluded that exists an important collateral communication between the cremasteric and deferential vessels at second-stage FS procedure (41).

Braga et al. (42) showed that the gubernaculum sparing laparoscopic orchiopexy is a feasible alternative to conventional laparoscopic Fowler-Stephens orchiopexy. This study concluded that this technique preserves an additional vascular supply to the testis (cremasteric, vessels and deferential arteries) during the laparoscopic orchiopexy.

Orchiectomy is adopted in cases of unilateral cryptorchidism in which the affected testis is atrophic, there is atresia or small length of the vas deferens, the testicular veins are located in the retropertium because they are too short or in the presence of intra-abdominal testis in the postoperative pubescent patients, associated with the contralateral testis without anatomical and morphological alterations (36). However, in post-pubescent children, there is controversy regarding the adoption of this approach as it would impair the patient's quality of life and increase the risk of postoperative mortality, with consensus in cases of increased risk of testicular neoplasia (43, 44).

## CONCLUSIONS

Preservation of an adequate arterial supply for the testis is crucial for successful orchiopexy to ensure normal testicular size and texture so the anatomy of testicular arteries is very important to success of the surgical procedures. Laparoscopic transection of the testicular vessels by dividing the spermatic vessels (Fowler-Stephens surgery) is safe in patients with high abdominal testes because the great collateral vascular supply between testicular, vasal and cremasteric arteries, and two-stage Fowler-Stephens orchiopexy appears to carry a higher rate of success than the single stage approach.
